# Abstracts and Gleanings

**Published:** 1877-10-20

**Authors:** 


### ABSTRACTS AND GLEANINGS.

MENOPAUSE.—Wm. Pepper, M.D., in University of Pennsylvania Clinic, remarks: Ovulation fixes woman's place in the animal economy. With the act of menstruation is wound up the whole essential character of her system. At the "change of life" we recognize a transformation in woman's moral, emotional and sexual character. There is no period of life, except that of early childhood, in which such peculiar conditions arise.

Let us consider the symptoms in order.

I. *Hemorrhages.*—Some mode of establishing an equilibrium is necessitated upon the stoppage of the natural menstrual flow. The circulatory fluid is in excess and must find some vent, hence piles, copius epistaxis, hæmatemesis, and hæmoptysis as resultants.

II. *Dyspeptic Symptoms.*—These are very varied, troublesome and hard to relieve. Women at this time are apt to overeat themselves. The same amount of blood-making food is consumed, and less blood is needed. Therefore, we find (1) actual indigestion, or (2) plethora. There is, in the first case, marked predominance of gastric nervous symptoms, such as heartburn, gastralgia and acidity, headache, disturbed vision and pains over the body, paroxysms of neuralgia, it may be. Vomiting is not common. Reflex disturbances, such as fixed pains in the head, are usual. The tongue is large and flabby, the liver congested and inactive. The complexion and conjunctiva may be sallow. Eruptions, such as acne, and crops of pimples arise. The bowels are torpid, and so a chronic catarrh of the intestines may be set up. The second set of symptoms are connected—the assimilation of organic matter into the blood. It may add to the mass of the blood and develop plethora, or it may form a low grade of tissue and cause obesity. Such persons suffer from a great sense of fullness and pulsation. The vessels of the head and neck beat and throb, particularly on bending down; there is giddiness and possibly confusion of mind and loss of memory. The pulse is full, hard, sounding and slow; the urine high colored, and contains an excess of organic matter. There may be oppression of the chest and fulness at the base of the brain. Polysarca comes on. The woman increases to double her former weight in the course of a few years.

III. *Moral and Emotional Changes.*—Women may become raving maniacs until the balance is restored. They grow nervous, fidgety, petulant, and hard to get on with. They indulge occasionally in the most peculiar and unnatural freaks.

IV. *Disturbances of Circulation.*—Palpitation and functional derangements are common. The pulse is frequent and irregular; there is often violent dyspnœa. No valvular murmur or hypertrophy exist.

V. Latent disposition to disease is exceedingly likely to be roused and become evident at this time. This is particularly the case with gout. The lithic acid diathesis is very common in this country, though gout is rare. The nitrogenous matters are only carried to the stage of uric or lithic acid, and these circulate in the blood, taking the form of calculi, and being deposited in the glands. All the conditions necessary to the uric acid diathesis are met with in the menopause. Such women are very likely to have gravel in the urine, thickenings about the joints, etc., etc. Other constitutional affections, such as cancer, phthisis, may also make their appearance now.

As regards diagnosis, it is only necessary to note the age of the patient, the menstrual irregularities, and the absence of any other cause for her disease.

The prognosis is generally favorable if the proper medicinal, moral, and hygienic treatment be employed. Otherwise softening of the brain, a permanently congested liver, diseases of the heart, and often incurable constitutional affections, are the inevitable consequences.

As regards treatment. The diet must be restricted, and all stimulating foods avoided, though the patient may feel weak and indisposed to exertion. Alcohol, organic foods and fats are to be strictly forbidden. So, also, with butter, meat and fried food. What is eaten should be light and digestible. Moral suasion is of essential importance. Explain to the patient the change she is going through, and so gain her confidence. Exercise, fresh air, sunlight, gymnastic pursuits, bathing, and horseback riding are the chief hygienic indications. If the patient be feeble, with a tendency to tuberculous diathesis, be careful how you put her on an exhausting treatment. Where the liver is congested, occasional moderate doses of blue pills are of benefit—3-5 grains once every fourteen days. I have seen the most wonderful results obtained under this treatment, especially if there be an excess of lithic acid in the blood, as shown by the red brickdust sediment in the urine. Some patients cannot take blue pills without salivation, so never give more than two grains at a time, followed by a saline. If calomel is not borne, Epsom or Rochelle salts may be substituted. So some bitter waters may be drunk after breakfast. For the nervous symptoms the bromides are the best remedies. Assafœtida and valerian are also useful. I have had very excellent results from two-grain doses of the monobromide of camphor, taken every two hours until ten grains have been ingested. Dry cups, mustard plasters, and blisters are of value as local applications. The symptoms of circulatory disturbances are to be best combated with digitalis. If, however, there be marked plethora, with a heavy, driving pulse, aconite or veratrum viride may be employed. For the indigestion the diet should be carefully studied. Sometimes the alkalies and nervines are needed on account of the peculiar accompanying pains. The following formula is reliable:

R. Acid. hydrocyan dil......gtt. i.
Sodæ bicarb................gr. x.
Tinct. valeriani.........fl dr. ½
Zingiberis syrupi..........gtt. xl.
Aquæ.......................fl. oz. iij.
S. In water, thrice daily.

Lithia, being a fixed alkali and good diuretic, is often employed with advantage. It has a tendency to eliminate the urates from the blood. The benzoate, carbonate or citrate of lithia are also employed. Where there is presumably a tendency to the development of some latent constitutional affection, it should, if possible, be anticipated and aborted. —*Medical Record.*

Amputation of the Arm by Means of the Elastic Ligature. — In the *Lyon Medicale* this operation is recorded as performed by Prof. O. G. Silvestri, of Vicenza. Surgeons naturally hesitate to perform resection or amputation in cases of white swelling of the knee or elbow. The process not being arrested on account of inadequate remedial measures, the patient loses strength, and becomes extremely emaciated; it is at this period of the disease that the operation is usually performed, though the general condition of the patient would almost contraindicate any active interference.

Silvestri, who first introduced the elastic compression known under the name of "Esmarch's method," has proposed the employment of the elastic ligature in the above cases, and has published a case in which the result was most gratifying. It was that of a young man, twenty-two years old, of a scrofulous constitution, who for six months had suffered from caries of the sixth, seventh, eighth and ninth ribs, in their convexities; there was complete caries of the left elbow-joint, and the right hand was threatened with the same condition. There were high fever, colliquative sweats, and diarrhœa, which would yield to no treatment; absolute anorexia, intense pains in the elbow, and extreme emaciation. Though the condition of the elbow-joint indicated an operation, the feebleness of the patient contraindicated it. But, as the patient was urgent to have something done, Silvestri, with the consent of his colleagues, resolved to apply the elastic ligature.

On the 8th of May, 1874, accordingly, the patient's arm, below the insertion of the deltoid, was enveloped with a gum-elastic band, about two millimetres in diameter, and covered with silk thread. Twenty turns of the band were made, the latter being always kept in its greatest extension, and the two ends were tied with a silk band. The patient received seven and a half grammes of chloral, which produced sleep. No pain was experienced. The pressure exercised, calculated according to the elasticity of the band, was equal to twenty one killogrammes at each point, consequently forty-two killogrammes for the whole diameter. The pulse, at time of operation, was 100; five hours after, 112; and six hours after, 100. There was no fever on the following day; the sweats and diarrhœa ceased, and the appetite returned. Milk diet was ordered, under which the patient soon began to gain flesh.

Gradually the bands penetrated the soft tissues, and at the same time lost their parallelism. The circumference of the arm, where the bands were applied, was eighteen centimetres at the time of operation; four days after, it was eleven centimetres; six days after, ten and a half centimetres, and ten centimetres on the 26th of May. On the evening of May 29th, it was found to be nine and one-quarter centimetres, and on June 3d it was reduced to eight centimetres.

On June 18th the arms and bands fell off spontaneously, the process having lasted forty days. The stump, in its upper portion, had cicatrized. The remaining portion was dressed with dry lint. The further course of the case was favorable.

The author draws the following conclusions:

1. The compression exercised intercepts all communication between the limb and the rest of the body; the morbid material from the seat of the disease cannot, therefore, enter the circulation; furthermore, drainage from the morbid *foyer* ceases.

2. There is no loss of blood.

3. Cicatrization takes place slowly, and the patient bears it easily.

4. The patient's forces are economized.

The author does not hesitate to employ this method in all those cases where the general condition of the patient offers no prospect of success to the performance of a bloody operation.—*N. Y. Medical Journal.*

VALUE OF SMALL DOSES OF MEDICINE FREQUENTNY REPEATED.—We cull from the *Medical Record* the following practical items, from a paper read before the New York *Medical Journal* Association, by Dr. Henry Dessau:

VOMITING IN CHILDREN.—In the treatment of vomiting in children, whether due to stomach and intestinal disorder, or as a complication of pneumonia, following the recommendation of Ringer, I have found the administration of drop doses of the wine of ipecac, repeated every hour, act with the greatest success in checking the vomiting. It also appears to exert a curative effect upon the diarrhœa of children when attended with vomiting, especially that form where the stools resemble those of dysentery. But the vomiting is the symptom that is most markedly benefitted. I recall the case of a little patient at the New York Foundling Asylum, suffering from a severe attack of croupous pneumonia, where the stomach was so irritable for the first two days of the attack that not even a spoonful of toast-water would remain. This condition, of course, prevented the retention of any remedies, but after the first dose of a drop of wine of ipecac given in toast-water, the nurse reported that the vomiting entirely ceased and did not return. The remedy was, however, continued for two days longer. Frequently, where other remedies would not be retained in the vomiting of children suffering from acute gastro-intestinal catarrh, the drop dose of wine of ipecac, given in toast-water or the mother's milk, would remain and quiet the stomach to receive other remedies, and, most important of all, the mother's milk.

VOMITING AFTER DEBAUCH.—In the vomiting sometimes following a debauch, especially in women, of which I have seen several severe cases, drop doses of Fowler's solution of arsenic, hourly repeated, appeared to act like a charm. This remedy is also highly recommended by Ringer in the morning vomiting of drunkards, where this symptom is indicative of a chronic affection of the gastric mucous membrane. Here the dose is not so frequently repeated, however, a drop of the solution given three times daily, before meals, being sufficiently often. Where there is a disgust for food, in addition to the morning vomiting, in these cases of chronic alcoholism, I have used a combination of a drop of Fowler's solution of arsenic and from three to five drops of tincture of capsicum, given before meals three times daily, with good success.

VOMITING IN PHTHISIS.—In the vomiting which often complicates phthisis pulmonalis and its allied affection, chronic bronchitis, independent of that brought on by the cough, it is of the utmost importance to be possessed of a reliable remedy to check it. The power of the stomach to retain its contents is here the only hope of any chance to strengthen and build up the general system, and so enable it to resist the encroachments of the disease-action. Moreover, the frequent attacks of vomiting tend to exhaust and weaken an already feeble organism, and to hasten dissolution. It is almost astonishing to observe with what happy success small doses of alum, say from three to five grains, given in solution with some aromatic water, as cinnamon for instance, acts here.

BRONCHITIS OF CHILDREN.—There is a form of bronchitis seen amongst children, where a large number of coarse mucous rales produce loud wheezing with an asthmatic quality of cough. The wheezing is the symptom that the mother is more likely to complain of, and together with the cough, is most intense at night, both almost entirely disappearing during the day. Such cases very readily yield in my praetice under the use of tartar emetic, given in solution in the proportion of a grain to the pint of water. Of this solution a teaspoonful is given every one or two hours, with the best results, sometimes, according to Ringer, relieving the noisy wheezing after one or two doses.

Often in children we find a catarrh of the bronchial and intestinal mucous membranes, either coexisting or alternating with each other. When such a condition persists after the employment of the ordinary household remedies, tartar emetic, in the same doses of the solution just before mentioned, hourly repeated, will check both catarrhs without the use of further treatment. This plan is, at least, an advantage over the usual one of prescribing separately for the cough and diarrhœa. I am indebted to Ringer for the suggestions of the foregoing treatments.

ALIMENTATION OF INFANTS WHILE SUFFERING FROM INTESTINAL CATARRH.—Prof. Demme, of Berne, believes that cow's or goat's milk should not be given at all to infants who are suffering from intestinal catarrh. It appears in the passages in the form of coagulated, undigested masses. The affection prevents the formation or the normal action of the gastric ferment, and the milk, in traversing the digestive canal, acts as a foreign body, which irritates the mucous membrane, undergoes a putrid fermentation, and causes the continued formation of watery, putrid discharges. As a rule, human milk does not exert these unpleasant effects. Condensed milk is less injurious than cow's milk, although the large quantity of sugar it contains favors fermentation and decomposition. In the Hopital Jenner, infants affected with acute intestinal catarrh are nourished in the following way:

Twice a day from a quarter of a pound to a pound of beef is deprived of fat, cut up into fine pieces, infused for an hour in a quart and a half of cold water, and then boiled down to a quarter of its previous bulk; it is then set aside for a while, the fat skimmed off, and the remainder filtered and set aside to cool completely. It is given cold, in small quantities, every two or three hours, and mixed with barley or rice water, which should be prepared first each time by boiling a small quantity of rice or barley in water for a few minutes. When the children are very thirsty, the rice or barley water is given in the intervals without sugar. When they refuse to take the water and bouillon, Professor Demme resorts at once to albuminous water. This is prepared by beating up the whites of one or two eggs, and then adding water in small quantities at a time, while the mixture is being gently stirred. In this way a well mixed fluid is obtained, which is mucilaginous and tasteless, and is readily taken by the children. For infants from one to ten weeks of age, Prof. Demme orders for the bouillon from a quarter to a half pound of meat per diem. One, two or three whites of eggs, according to the age of the patient, suffice for from half a pint to a pint and a half of water, which quantity may be administered daily. When the patients begin to lose strength, he gives from five to thirty drops of the purest cognac from three to five times a day. When, in the cases of older children, it is deemed advisable to continue the use of milk, it is diluted with rice or barley water. He has given up the use of grated raw meat in acute intestinal catarrh of infants.

With regard to medicinal treatment, Prof. Demme has found that the profuse serous diarrhœas yield readily to very small doses of calomel (gr. 1-20 to 1-10) and opium (gr. 1-100 to 1-35) every two or three hours. Sometimes has had recourse with advantage to nitrate of silver, with glycerine and a small quantity of opium. He leaves out the opium when possible, because it lessens the strength of the patients. He believes that tannin, bismuth, the vegetable astringents, etc., only irritate the mucous membrane and prolong the affection. The above plan of nourishment is as applicable for the chronic as for the acute form of intestinal catarrh. In the former, Mr. Demme has found the continued use of albuminous water and cognac to be especially valuable.—*Gazette Obstetricale,* April 5, 1877.

Dose and Mode of Administering Nitrate of Silver.—Many years since I began to administer this remedy, giving a grain in an ounce of pure water in the morning before breakfast, for chronic inflammation of the stomach and bowels. I have continued it with the most gratifying results in dyspepsia, with its various morbid symptoms. As I don't weigh it any longer, I have given, no doubt, one and a half grains per dose at times; I have weighed five grains and divided it into four doses, often. I find it sometimes vomits, sometimes purges actively, and sometimes produced no decided sensation. But it is the best alterative and tonic to the mucous membranes of the stomach and bowels I have ever tried. I give it alone for that. I generally give a dose every other morning, until two, three or four doses have been taken. I always put it up in blue paper, to be dissolved in four tablespoonfuls of rain water, when used, and drank on an empty stomach before breakfast, always. In this way and for this purpose I use a great deal, with results highly gratifying to my patients as well as myself.

I have been induced to give this, my experience in the use of nitrate of silver, from noticing several articles on the subject in the *Reporter.* I have acquired quite a reputation for curing epilepsy. I give nitrate of silver (with other remedies) simply to restore the mucous membranes of the stomach and bowels to a healthy condition. I never continue it long; seldom give more than four to six or eight doses; when I give more than four, I leave considerable interval between the first and second course. I don't depend upon that article alone in the treatment of epilepsy.—*D. W. Foster in Med. and Surg. Reporter.*

[It should be borne in mind that this remedy will discolor the skin if long continued.—Ed.]

Autumnal Diarrhea.—The season is approaching during which the common autumnal or summer diarrhea is one of the most common complaints for which the physician has to prescribe. As most physicians are aware that a vast amount of it is caused and kept in action by an acid state of the stomach and indigestion, and that laxatives and antacids will generally control it, I present the simple and inexpensive form which I have employed for the last five years with entire success. It will sometimes, though rarely, be necessary to employ a little hydrarg. cum creta or quinine in connection with it.

R Pulv. rhei.......... }

Magnesiæ.......... } *aa* gr. xl;

Sodii bicarb........ }

Sacchari alb.... ... oz. ij;

Ol. anisi............ gtt. xl;

Aquæ................ fl.oz. viij;

Tinct. opii camph fl.oz. ss.

Drop the oil of anise on the sugar in a mortar, add the powders, and mix gradually; add the water, pour all into a bottle, and add the paregoric. Shake well before using.

Dose for infant, one half teaspoonful; one to two years old, one teaspoonful; two to ten years old, two teaspoonsful; adults one or two tablespoonsful, from three to six hours apart. If it should be necessary to use an astringent, as dry chalk,

R Cretæ prep......... oz. j;
Pulv. kino........... dr. j. M.

may be prescribed in doses sufficient to produce the desired effect.

This will be all that will be required in most cases of our fall diarrheas, especially among children, and it has the advantage of being easily prepared and pleasant to take.—*Dr. W. McWilliams, in Ohio Med. Recorder.*

USES OF HYDRATE OF CHLORAL.—Dr. J. A. Larrabee, (Va. Med. Monthly) says: I have found chloral hydrate, in strong solution, to be the most agreeable disinfectant and reliable antiseptic. Administered in febrile conditions it is an apyretic of no mean value; and applied locally in union with camphor or in strong solution, it is a descutient, anodyne, and alterative of great power. It forms the best application for injection into suppurating sinuses, as in the hip, long muscles of the back, etc., cleansing and healing rapidly these fistulous tracts and promoting the rapid healing of superficial ulcerations.

It will arrest the formation of *furuncles, carbuncles,* and cause rapid *resolution of indolent* swellings. A number of children were selected suffering from most *offensive ozœna* and strumous sores. In less than a week all fetor ceased and *recovery* followed the use of a mild solution with the nasal syrine.

The most wonderful result was obtained in a lot of children suffering from scorbutic gums. Six of the worst cases were selected; in some of them, the gums were sloughing, and all presented a frightful appearance. The local use of chloral hydrate in water as a wash cured these gums in eight days—sound and well—constitutional treatment being kept up during the mean time. It is necessary, however, to state that the same constitutional treatment had been kept up without benefit for a month previous. It has also been used, with great advantage in ptyriasis copitis.

STRINGENT AND TONIC BATHS IN SUMMER COMPLAINT OF CHILDREN.—Dr. Wharton (Va. Med. Monthly) says: Many a time, when the case seemed almost hopeless, have I seen the baths of oak and willow bark apparently snatch the patient from the very verge of the grave, and it is from an experience of its great value in such cases—an experience of thirty years—that I would emphasize their importance.

I do not undervalue the importance of medicine when I say that I have seen more good done in bad cases of summer complaint by the use of the baths than by all other means. During the present season I have seen some very bad cases relieved promptly, after the failure of well-directed treatment with the established remedies.

The baths are prepared by boiling oak and willow barks to a strong decoction. The patient is bathed in this decoction at a temperature of 80° F.—that is, tepid—as a warm bath is too relaxing, and he will not be able to bear a temperature cold enough to cause a shock. He must be be kept in it from five to ten minutes, according to his strength and susceptibility, and it must be used twice a day in severe cases. The same water will be good for two days in the hottest weather.

ARSENIC IN ALBUMENURIA.—Dr. T. Lauder Brunton reports the case of a man who, for nine years, suffered from intermittent albuminuria, with debility, and at times loss of flesh. The albuminuria was worse in summer, was brought on by exertion in the morning, and by the use of meats and fats, and completely disappeared when a farinaceous diet was adhered to. The patient suffered much from acidity, especially in the morning. There was no œdema. The heart and lungs were healthy. No casts were found in the urine, but it was not very frequently examined for them. Quinine increased the albumenuria, and so did phosphorus. Digitalis caused it to diminish slightly, but the drug disagreed with the stomach, and had to be stopped. On account of its supposed action on the secreting structure of the kidney, Fowler's solution was finally ordered at meal times in three-minim doses. The albumen disappeared from the urine almost at once, and the patient was able to do much more work than before without bringing it back.—*The Practitioner, June,* 1877.

A Daring Therapeutist.—At a late meeting of the Massachusetts Dental Society, Dr. Waters, of Salem, stated that bicarbonate of soda, such as is used for cooking purposes, or any other alkali in neutral form, would afford instantaneous cessation of pain from the sèverest burns or scalds, and would cure such injuries in a few hours. Dipping a sponge into boiling water, the Doctor squeezed it over his right wrist, producing a severe scald around his arm and some two inches in width. Then, despite the suffering it occasioned, he applied the scalding water to his wrist for half a minute. Bicarbonate of soda was at once dusted over the surface, a wet cloth applied, and the pain, the experimenter stated, was almost instantly deadened. Although the wound was of a nature to be open and painful for a considerable time, on the day following the single application of soda, the less injured portion was practically healed, only a slight discoloration of the flesh being perceptible. The severer wound, in a few days, with no other treatment than a wet cloth kept over it, showed every sign of rapid healing. —*Med. and Surg. Journal.*

Reduction of Heat in Typhoid Fever—Dr. Kibbee (*Medical and Surgical Reporter*) states:—I have proven, to an absolute certainty, that the pouring of tepid water through a sheet, so folded that it will reach from the lower part of the hip to the axillæ, will, in a short time, two hours at most, reduce the highest fever heat to the normal standard, and hold it there for days, and even weeks, as in many cases of typhoid, which I have treated with invariable success, where the treatment began with the first exacerbation of the fever.

In my time I have had several calls to sit in council with my medical confreres who had treated their typhoid cases in the usual way almost up to the period of *articulo mortis*, a point past all hope from the use of any medicinal remedy known to the profession; and by simply adopting the tepid pouring process, the fearful internal heat was reduced to the normal standard, and held there by continual pouring, and equalized by applying artificial warmth to the extremities.

Chronic Malarial Toxemia.—Having been a busy practitioner in the swamps of Louisiana for the last six years, where I have been called upon to prescibe for all the varied and masked forms of chronic malarial poisoning, I have found nothing so efficient and efficacious as an alterative and tonic, as well as a permanent curative agent, as the following prescription, which, if continued for a sufficient length of time, will do all that a combination of medicines can do in such cases; in fact, no case coming under my observation, during the past six years, has resisted this treatment, and am satisfied no one will be disappointed who will give it a thorough trial:

R. Pulv. sulph. ferri.......... 4 drachms.
Nitric acid................ 1 ounces.

Mix and stir in mortar till effervescence ceases then add:

Sulph. quinine.............. 4 drachms.
Nitrate potass............ 6 drachms.
Aqua Q. S. to make...... 6 ounces.

S. Teaspoonful three times daily, after eating, well diluted in water.

Lake Charles, August 8th, 1877.

—*Brief.*

Sexual Hygiene.—Professor Hartshorn, formerly Professor of Hygiene in the University of Pennsylvania, gives the following condensation of his views of sexual hygiene:

1. The action of the reproductive organs is not necessary to the individual; its purpose being the continuation of the species.

2. No harm results from inaction of the generative organs.

3. Sexuality is healthy and safe only in marriage.

4. The married state is, as a rule, rather more favorable to health than celibacy.

5. Abnormal sensuality is injurious in proportion to prematurity, deviation from nature, and frequency of indulgence.

6. Hygiene furnishes no justification for prostitution.—*Med. and Surg. Journal.*

INFLUENCE OF ARTIFICIAL SUPPRESSION OF THE CUTANEOUS SECRETION.—Sokoloff (*Virchow's Archiv.*) experimented upon some forty-six dogs and rabbits. These animals were painted with oil and other substances, the results being as follows: The temperature usually fell. The urine showed gray and hyaline cylinders, kidney-epithelium, and young cells by which its specific gravity was increased; albumen also appeared. In one case, dropsy occurred. Diarrhœa, loss of power in the heart, cramps, sopor, and, when the coating was extensive or complete, death, in periods varying from a few hours to several days. Post-mortem examination, congestion of the brain and membranes, as well as of most of the internal organs. —*The Drug. Cir. and Chem. Gaz.*

INSOMNIA.—A lecturer remarks that ordinary cases of insomnia may be divided into three classes—senile, toxic, and psychical. In the senile form of the affection the disorder depends upon degeneration of the cerebral arteries, and is difficult of cure; in the toxic, upon abuse of alcohol, tea or tobacco, and ceases upon the removal of the cause; in the psychical, it arises from continued and excessive mental strain, grief, anxiety, worry, etc., and is usually successfully treated by full doses of bromides, combined with tincture of ergot and cod-liver oil. If the insomnia be serious, it must be stopped at once by hypnotics, preferably by opium.—*Med. and Surg. Reporter.*

BELLADONNA IN CONSTIPATION, THE RESULT OF PARALYSIS OF THE BOWELS.—Dr. H. A. DuBois, of San Rafael, Cal., writes: "In a case of paralysis of the bowels, the result of apoplexy, where there had been no discharge for two weeks, and where croton oil and other powerful cathartics had failed, and in which the ascending colon only could be emptied by the rectal tube, small doses—five drops three times a day—of the fluid extract of belladona, continued for two days, enabled the tube to enter the transverse colon, and procured thorough action through the whole length of the canal. I was indebted to Dr. Taylor, U. S. Navy, for this suggestion."

TREATMENT OF NASAL CATARRH.—Dr. Hartmann, of Berlin, recommends the use of Politzer's method for distention of the middle ear, in the treatment of acute nasal catarrh. By the compression of the air in the nasal cavities, the collected secretions in the frontal sinuses and other cavities opening into the nasal fossæ, is forced out, and the pains and other disagreeable sensations in the head are thereby greatly relieved. In order to prevent any undesirable effects on the middle ear, the external auditory canals should be closed with the fingers, whereby a too forcible driving outwards of the drums is prevented.—*Memorabilien,* Heft 6, 1877.

POST-PARTUM HEMORRHAGE.—Dr. Brisbane (*London Lancet*) gives the following treatment: Nothing more is required than to carry a two ounce bottle of ordinary tincture of the muriate of iron. A piece of sponge can always be obtained. This is compressed in the palm of the hand and the iron poured on it, and thus it is conveyed up the bleeding surface of the uterus, against which it is pressed and left *in situ.* The blood coagulates, the uterus contracts, and the patient is rescued from the most imminent danger. I have have at my next visit always found the sponge in the vagina, nor have I ever seen any bad effects follow.

ANTISEPTIC VINEGAR.—The following formula for antiseptic vinegar is given by M. Pennes:

| | | |
|---|---|---|
| Salicylic acid | 300 | grammes. |
| Acetate of alumnia | 300 | " |
| Tincture of eucalyptus globulus | 1,000 | " |
| " verbena | 900 | " |
| " lavender | 1,000 | " |
| " benzoin | 100 | " |
| Acetic acid ("No.8") | 1,000 | " |

Mix thoroughly with the requisite precautions, shake frequently during two or three days, and fiiter. This vinegar may be used either pure or diluted as a lotion. Five litres injected into the carotid will preserve a cavader several months. In the form of spray it is recommeded as an admirable disinfectant for the sick-room. —*New Rem.*

Extraction of Foreign Bodies from the Œsophagus in Children.—In allusion to a case in which there had been some difficulty in extracting a coin swallowed by a child, Dr. Thouvenin states that in such cases he adopts a very simple measure with great success. It consists in laying the child flat on his belly, on a table, with his head, supported by an assistant, projecting beyond it. The finger is then introduced into the mouth in order to depress the tongue, and the coin slides out along the finger of the operator.—*Bull. de Therap.*, *Medical Times and Gazette, April* 14, 1877.

Lasinski recommends a powder made of dr.ss. salicylic acid, grs.xv. quinine, and grs. viij aa of sugar and bicarb. soda for pertussis. The whole quantity should be so divided that it would last ten days, using it twice daily. Each part should be blown into the throat with an insufflator. After each insufflation a certain degree of suffocation follows which soon passes by.

Under this treatmnt the writer had cured more than fifteen cases of pertussis convulsion, between the fifth and thirtieth day.—*Centralblatt.*—*Maryland Medical Journal.*

Podophyllin in the Treatment of Hepatic Colic.—In a paper published a few months ago in *Lo Sperimentale*, Prof. Bufalini reported two cases of severe hepatic colic that were cured by the use of small daily doses of podophyllin. Prof. Bufalini ordered small doses of podophillin (gr. one-sixth per diem), and both the hepatic colic and the intestinal catarrh rapidly disappeared. The use of podophyllin was continued for a year. and, during that time and the two years that have since elapsed, the colic did not return.—*Gazette des Hopitaux*, July 7th.

Cosmoline is one of the products derived from petroleum after the more volatile portions have been removed by distillation. It has none of the odor or taste of the oil; it is semi-solid, and may be melted by heat. As it does not oxidize or become rancid, and is uniritating, it is an admirable vehicle with which to prepare ointments—far better than lard or any of the cerates. Or mixed with the ordinary officinal ointments it prevents them from changing or spoiling.

Iodoform in Ear Diseases.—Dr. Rossett, of North Carolina, (in *Maryland Medical Journal*) calls attention to the excellent effects of iodoform in inflammation of the tympanum and eustachian tube, particularly where there is perforation. Cleanse the ear by injections of tepid water, then dry with pledgets of cotton on a probe, and apply the iodoform on a little mop in quantity sufficient to cover the tympanum or the internal diseased surface.

Chloral—How Prepared.—Chloral was discovered more than thirty years ago by Liebig, but was not used medicinally until as late as 1869. It is produced by the action of chlorine on alcohol. Alcohol yields 5 eq. of its hydrogen to the chlorine, forming 5 eq. muriatic acid, and combining in their place with 3 eq. of chlorine, to form chloral.

Poisoning by Salicylic Acid.—A case of poisoning, resulting in death, is reported in a German journal, from four doses of salacylic acid, twelve grains each, given at intervals of an hour. The preparation used was old, and supposed to have undergone chemical change. The pure crystaline article should only be used.

Sulphophenate of Soda.—M. Brakenridge has employed this salt in scarlet fever in fifty cases, all of which were cured save three, who entered the hospital at too advanced a stage of the disease. The dose is from 15 to 25 grains. —*Lyon Medical*

Diabetes.—Salicylicate of soda has been found useful in diabetes.

TREATMENT OF BLENNORRHAGIC EPIDIDYMITIS WITH IODOFORM OINTMENT.—Dr. Alvares, of Palma (Majorca), has treated four cases of epididymitis with iodoform ointment, and from his experience in these cases draws the following conclusions:

1. Iodoform calms the pain of blennorrhagic orchitis better than any other application; this result is obtained at the end of one or two hours.

2. Iodoform exerts a very manifest resolvent action, and has the advantage over the usually employed mercurial ointment, of causing no trouble when absorbed.

3. The iodoform treatment shortens very appreciably the duratiou of the orchitis, and prevents any consecutive induration of the organ.

4. It is necessary to employ an ointment containing, according to the intensity of the inflammation, from one to two grammes of iodoform to thirty grammes of lard.—*Le Bordeaux Medical* June 26th.

ERGOTIN IN METRORRHAGIA.—The Philadelphia *Medical Times*, May 12, translates two cases from *La France Medical*, March 31, of uterine hemorrhage cured by injections of ergotin into the subcutaneous cellular tissue of the arm. The solution was made by dissolving 15 or 20 grains of ergotin in a drachm of a mixture composed of water, glycerine, and alcohol. In the first case the quantity of ergotin given is not mentioned; in the second, 12 grains were given the first day, and 18 grains on the second, which put a stop to the hemorrhage. This, however, returned after an interval of ten days; the injections were repeated, and removed the trouble, which did not recur.

IDROSIS.—Dr. A. L. Sweet, of Long Island, writes: I have never noticed, in the medical journals which have come under my observation, anything in regard to the use of the juniper tar soap in excessive sweating of the feet. If it is not generally known, it may be of interest to state that I have found nothing comparable to the above named remedy for this distressing and frequently disgusting affection. I use that prepared by Caswell & Hazard, and have recently completely relieved the worst case I ever saw (the patient my brother-in-law), where the secretion was so abundant and offensive as to destroy a pair of shoes in a short time, and fill nearly the whole house with the odor.—*Med. Rec.*

FEVER AND AGUE.—The following prescription for fever and ague has never failed in my hands to afford prompt and permanent relief:

| | | |
|---|---|---|
| R. Cinchonidiæ sulphas........ | | grs. lxiv. |
| Acid sul. aromat........... | | q. s, |
| Tinct. nucis vom. ........ | | m clx. |
| Syrup simplici.... .....ad. | | f. oz. iv. |

M. One teaspoonful every four hours until the paroxysms are broken, then two doses a day for a week or more. This seems to be a small dose of the alkaloid, but in the combination it is perfectly effectual, produces no unpleasant head symptoms, and the patients are not nearly so subject to relapses as when the quinine sulphate is used. —*Ibid.*

TREATMENT OF FACIAL NEURALGIA.—At the Presbyterian Hospital, New York City, nothing has been found so satisfactory as croton chloral, in the treatment of neuralgia, migraine and sick-headache. It is given in solution with elixir calisaya; five grains, three times a day, for a week. When taken in this manner the habit of the neuralgia is broken up, and permanent cure frequently results.—*New York Med. Record*, May 5.

PHOSPHIDE OF ZINC will be found serviceable in defective nutrition of the brain, spinal irritation, hysteria, and in various forms of paralysis.

VASELINE is supposed to be obtained from petroleum in a manner similar to cosmoline, and possesses a like chemical nature.

